# Response to Mooij and van Trotsenburg re: “Exploring the Genetic Link Between Thyroid Dysfunction and Common Psychiatric Disorders: A Specific Hormonal or a General Autoimmune Comorbidity”

**DOI:** 10.1089/thy.2023.0315

**Published:** 2023-08-07

**Authors:** Marco Medici, Sourena Soheili-Nezhad

**Affiliations:** ^1^ Department of Internal Medicine, Academic Center for Thyroid Diseases and Rotterdam, the Netherlands.; ^2^ Department of Epidemiology; Erasmus Medical Centre, Rotterdam, the Netherlands.; ^3^ Department of Cognitive Neuroscience, Donders Institute for Brain, Cognition and Behaviour, Radboud University Medical Centre, Nijmegen, The Netherlands.; ^4^ Language and Genetics Department, Max Planck Institute for Psycholinguistics, Nijmegen, The Netherlands.

**Dear Editor:**

Our publication in the February issue of *Thyroid*^1^ has led to a lively discussion on the relations between thyroid (dys)function, psychiatric disorders, and its potential genetic and autoimmune underpinnings. In an accompanying commentary Nygaard systematically reviewed pitfalls in available literature and knowledge gaps to overcome.^2^ In this issue, Mooij et al. specifically elaborated on a systematic review showing that a substantial part of the studies did detect an association between thyroid peroxidase antibody (TPOAb) positivity and quality of life (QoL)/persistent complaints, while also acknowledging that part did not.^3^ With these two commentaries the discussion shifted toward the clinical applicability of our findings, especially for the unsolved common problem of persistent complaints despite biochemical euthyroidism on LT4 suppletion.

As illustrated by the aforementioned review on TPOAbs and QoL/complaints, results from observational and clinical studies in this field and outcomes in clinical practice have one thing in common: large heterogeneity. This is most likely as it concerns subjective endpoints (persistent complaints such as tiredness) potentially caused by a multitude of factors, examples of which include coexistence of other autoimmune diseases (and related treatments), suboptimal LT4 titration, lack of endogenously produced T3 and/or genetic variants affecting T4 to T3 conversion, and potential direct effects of underlying (auto)immune pathways common to both thyroid and psychiatric disorders.

How to move forward? Decades of research has resulted in the identification of these potential causes of persisting complaints, but most studies have investigated these factors individually, while not taking the other factors into account. Furthermore, the far majority of studies has not been performed in the specific subgroup of interest, that is, dedicated cohorts of patients with persistent complaints.

Therefore, further substantial steps in this field can only be made with carefully selected dedicated cohorts of patients with persistent complaints with in-depth phenotyping of their mental and thyroid state, and potentially coexisting autoimmune diseases. Biobanking should enable extensive molecular and genetic analyses to identify subgroups, for example, patients in which the complaints are more driven by a lack of local T3 bioavailability due to a type 2 deiodinase polymorphism versus an increased (auto)inflammatory state of the brain. Clinical trials, such as the one Mooij et al. suggested in this issue,^3^ will benefit from this careful selection of candidates leading to a more homogeneous study population thereby decreasing the risk of false-negative results.

While easily written, it is beyond doubt that it will be a tremendous effort to collect such dedicated cohorts, especially since large numbers will be needed given the likely existence of multiple subgroups. This will require extensive collaboration and, preferably before collection, a consensus in the field on how such studies should look like. An elegant example of how the thyroid field has shown to be able to produce such a common view is the consensus document on design of a definitive LT4/LT3 combination trial, thereby ensuring widespread international support for its results.^4,5^

## Authors' Contributions

M.M. and S.S.N. drafted this response letter together.
